# Which strategy is better for linkage analysis: single-nucleotide polymorphisms or microsatellites? Evaluation by identity-by-state – identity-by-descent transformation affected sib-pair method on GAW14 data

**DOI:** 10.1186/1471-2156-6-S1-S16

**Published:** 2005-12-30

**Authors:** Qingqi Yue, Victor Apprey, George E Bonney

**Affiliations:** 1 National Human Genome Center, Howard University, Washington, DC 20059, USA; 2 Department of Community Health and Family Medicine, Howard University, Washington, DC 20059, USA; 3 Division of Medical Genetics, Department of Pediatrics, Howard University, Washington, DC 20059, USA

## Abstract

The central issue for Genetic Analysis Workshop 14 (GAW14) is the question, which is the better strategy for linkage analysis, the use of single-nucleotide polymorphisms (SNPs) or microsatellite markers? To answer this question we analyzed the simulated data using Duffy's SIB-PAIR program, which can incorporate parental genotypes, and our identity-by-state – identity-by-descent (IBS-IBD) transformation method of affected sib-pair linkage analysis which uses the matrix transformation between IBS and IBD. The advantages of our method are as follows: the assumption of Hardy-Weinberg equilibrium is not necessary; the parental genotype information maybe all unknown; both IBS and its related IBD transformation can be used in the linkage analysis; the determinant of the IBS-IBD transformation matrix provides a quantitative measure of the quality of the marker in linkage analysis. With the originally distributed simulated data, we found that 1) for microsatellite markers there are virtually no differences in types I and II error rates when parental genotypes were or were not used; 2) on average, a microsatellite marker has more power than a SNP marker does in linkage detection; 3) if parental genotype information is used, SNP markers show lower type I error rates than microsatellite markers; and 4) if parental genotypes are not available, SNP markers show considerable variation in type I error rates for different methods.

## Background

A key issue in nonparametric linkage analysis is the accuracy in the estimation of the relative pair identity-by-descent (IBD) distributions. The Genetic Analysis Workshop 14 (GAW14) simulated data provide an opportunity to evaluate new or existing methods for linkage analysis since the "answers" were known to the designers of the simulated data. We applied two types of methods to find the locations of linkage and determine the power and type I errors for single-nucleotide polymorphism (SNP) and microsatellite markers according to whether or not parental genotypes are available. The first method is the affected pedigree member (APM) method implemented in Duffy's SIB-PAIR program [1], which uses all the pedigree information including the parental genotypes and parental-sibling relationships and based on Weeks and Lange's method [2]. The second method is our recently developed IBS-IBD (identity-by-state – identity-by-descent) transformation method, which generalizes Lange's affected sib-pair method [3] and uses the affected sib-pair genotypes only. In this paper, we compared their power and type I error rates under the two different data assumptions when parental genotypes are available and when they are not available.

## Methods

We applied two types of methods to determine the performance (power and type I errors) for SNP and microsatellite markers with different data assumptions based on the availability of parental genotypes. The first method is the APM method implemented in Duffy's SIB-PAIR program, which was fully documented in [1]. The second method is our recently developed IBS-IBD transformation method, which generalizes Lange's affected sib-pair method [2] and currently uses only the affected sib-pairs. The method is based on the following proposition:

### Proposition

Assume that 1) parental mating is random; 2) in the parental population, for any genotype the two possible phase known genotypes have the same probability; 3) for each mating type that produces a sib-pair with IBD = 0, the two possible sib pairs have an equal probability to come; if the IBD = 1, the shared IBD allele has an equal probability to come from each one of the two parents. Let *P*_*ij *_(*M*) = *P*_*ji*_(*M*) = 1/2 of the sum of frequencies for the genotypes *a*_*i*_/*a*_*j *_and *a*_*j*_*/a*_*i *_in the parental generation with *a*_*i*_(*i = *1, 2, ...,*n*) being the alleles over the marker. Then in a full sib pair population without gender differences, the IBS and IBD probabilities are related by



where the transformation matrix *T *= [*T*_*ij*_] with *T*_*ij *_= *p*(*IBS *= *i*|*IBD *= *j*) 0 ≤ *j *≤ *i *≤ 2 is given by



*T*_11 _= *Het*(*M*)

*T*_21 _= *Hom*(*M*)

*Hom*(*M*) and *Hom*(*M*^2^) are the sums of all diagonal elements for the matrix [*P*_*ij*_(*M*)] and [*P*_*ij*_(*M*)]^2^, respectively, *Het*(*M*) and *Het*(*M*^*2*^) are the sums of all off-diagonal elements for the matrix [*P*_*ij*_(*M*)] and [*P*_*ij*_(*M*)]^2^, respectively, and *P*_*i*_(*M*) is the frequency for the *i*^th ^allele *a*_*i*_, (*i,j *= 1, 2, ..., *n*). The above formula reduces into Lange's [2] formula (with different form) for expected IBS distribution under the null hypothesis of no linkage and Hardy-Weinberg equilibrium assumption *P*_*ij*_(*M*) = *P*_*i*_(*M*)*P*_*j*_(*M*). Our formula can transform the IBD distribution to that of IBS by the transformation matrix *T *or vice-versa through the inverse transformation matrix *T*^-1^. With the estimates for IBD or the IBS probabilities, the statistics for nonparametric linkage analysis can be calculated and tested in the usual manner.

We performed all analyses without knowledge of the "answers." We still do not have the "answers," except those results appearing in the meeting abstracts.

## Results

**Figure 1:**
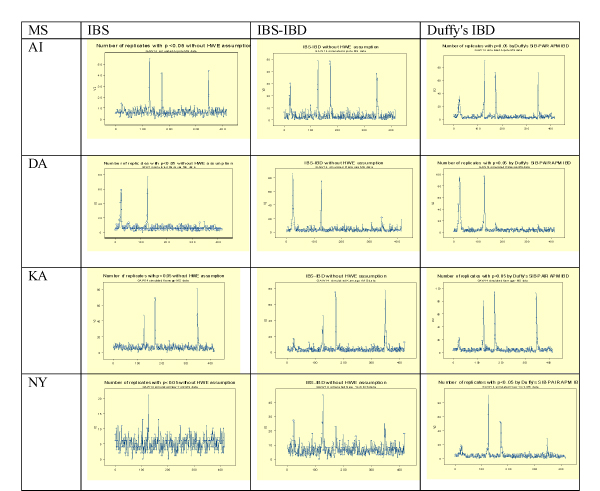
The number of significant (0.05 level) replicates over microsatellite markers.

Figure 1 provides the graphical comparisons of the performance of the SNP and microsatellite markers in the linkage analyses over the 10 simulated chromosomes, and for the four sites. We found four sharp peaks for the percentage replication rates at the 0.05 significance level in the linkage test over the 100 replicates at the following locations which were reported in our previous GAW14 meeting abstract: SNPs: C01R0052, C03R0280, C05R0380, and C09R0765 and microsatellites: D01S0023, D03S0127, D05S0172, and D09S0347. Our IBS and IBS-IBD methods detected an extra peak at SNP C10R0880 in the Danacaa sample.

To see the type I error rate, we listed the median of the numbers of significant replicates over all the markers, which could be viewed as an average type I error rate and graphically could be interpreted as the "noise level." Table 1 provides the information about the medians.

Based on our single-point linkage analysis, we observed the following results with respect to the comparison of SNP vs. Microsatellite markers (one SNP marker vs. one Microsatellite marker around the same location) and the effects of the parental genotype information in the comparison on the two types of markers.

1) On average, a microsatellite marker showed higher rates of significant replications than a SNP marker over the linkage locations.

2) Some SNP markers provided almost equally strong linkage evidence, for example, SNP C01R0052 in the Danacaa sample (see Figure 1, DASNP).

3) For the microsatellites, our affected sib-pair only methods showed a modest "noise" level (3%–6%) while the affected pedigree method (Duffy's APM) has a stable "noise" level of 3%. Both methods have almost the same "power." Since our method just used one affected sib-pair (no parental information), it seems therefore that parental genotyping may not be very critical in linkage analysis for microssatellite markers.

4) For SNP data, our affected sib-pair only IBS method has a "noise" level (4%–6%), IBS-IBD method has a high "noise" level (15%–21%) while affected pedigree method (Duffy's APM) has a stable "noise" level of 0%. The high "noise" level for IBS-IBD method reflects the fact that the IBS-IBD matrix for a SNP marker is close to singular. Thus, we conclude that for SNP data with parental genotypes, the false positive rate is very low in linkage analysis, and without parental genotype information the false positive rate can be relatively high.

## Discussion

The different sites vary with respect to the power to detect linkage. Since the linkage evidence over a marker for a disease is inversely proportional to the number of markers which interact in determining the phenotype, our results may reflect some characteristics of the four population groups. For example, the relatively weak linkage over the four locations in the Aipotu group (see Figure 1) is consistent with the fact that the affected Kofendrerd Personality Disorder (KPD) is defined by one or more of the three clinical categories: communally shared emotions, behavioral related and anxiety related, and the strong linkage over the first two locations (around D01S0023 and D03S0127) in the Danacca group (see Figure 1) may reflect the fact that only the behavioral symptoms were classified as affected in Danacca data. Similar results may reflect the characteristics in the other two populations.

## Conclusion

In summary, we conclude 1) for microsatellite markers there are virtually no differences in type I or type II error rates whether one uses or excludes parental genotypes. 2) On average, a microsatellite marker provides more power than a SNP marker does in linkage analysis. 3) If parental genotype information is used, SNPs show lower type I error rates than that of microsatellite markers. 4) If parental genotypes are not available, SNPs show variable type I error rates over different methods.

In summary, other things being equal in the simulated sample analyzed, microsatellites are better than SNPs, although if parents are typed, SNPs can have slightly better type I error rates.

## Abbreviations

APM: Affected pedigree member

GAW: Genetic Analysis Workshop

IBD: Identity-by-descent

IBS: Identity-by-state

KPD: Kofendrerd Personality Disorder

SNP: Single-nucleotide polymorphism

## Authors' contributions

QY developed the methodology, implemented the computer program, participated in the simulations and drafted the manuscript. VA participated in the simulations and GEB participated in analysis and coordination and helped to draft the manuscript.

## Figures and Tables

**Figure 2:**
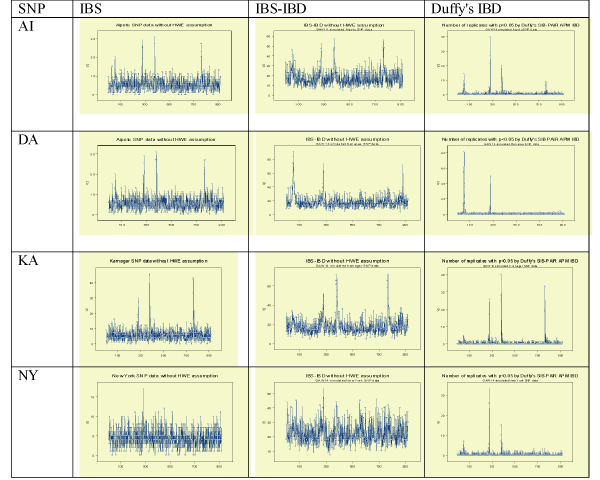
The number of significant (0.05 level) replicates over the SNPs.

**Table 1:** The medians of type I error rates over all the markers

IBS		IBS-IBD		Duffy's IBD	
SNP					
AI	5%	AI	15%	AI	0%
DA	5%	DA	16%	DA	0%
KA	5%	KA	16%	KA	0%
NY	4%	NY	21%	NY	0%
MS					
AI	6%	AI	3%	AI	3%
DA	6%	DA	3%	DA	3%
KA	6%	KA	3%	KA	3%
NY	5%	NY	6%	NY	3%
